# Addition of Standard Enzalutamide Medication Shows Synergistic Effects on Response to [^177^Lu]Lu-PSMA-617 Radioligand Therapy in mCRPC Patients with Imminent Treatment Failure—Preliminary Evidence of Pilot Experience

**DOI:** 10.3390/cancers14112691

**Published:** 2022-05-29

**Authors:** Florian Rosar, Hanna Bader, Mark Bartholomä, Stephan Maus, Caroline Burgard, Johannes Linxweiler, Fadi Khreish, Samer Ezziddin

**Affiliations:** 1 Department of Nuclear Medicine, Saarland University Hospital, 66421 Homburg, Germany; florian.rosar@uks.eu (F.R.); hanna_bader92@web.de (H.B.); mark.bartholomae@uks.eu (M.B.); stephan.maus@uks.eu (S.M.); caroline.burgard@uks.eu (C.B.); fadi.khreish@uks.eu (F.K.); 2 Department of Urology, Saarland University Hospital, 66421 Homburg, Germany; johannes.linxweiler@uks.eu

**Keywords:** enzalutamide, PSMA, upregulation, radioligand therapy, radiosensitizer, mCRPC

## Abstract

**Simple Summary:**

In this study, we investigated co-medication with enzalutamide, a well-established newer androgen axis drug, as a potential re-sensitizer for prostate-specific membrane antigen (PSMA)-targeted radioligand therapy (RLT) in n = 10 patients with imminent treatment failure on standard ^177^Lu-based PSMA-RLT. After the introduction of enzalutamide medication, all patients showed a PSA decrease (7/10 patients with partial remission). This pilot experience suggests the synergistic potential of adding enzalutamide to PSMA-RLT derived from the intra-individual comparison of ^177^Lu-based PSMA-RLT ± enzalutamide.

**Abstract:**

Well-received strong efficacy of prostate-specific membrane antigen (PSMA)-targeted radioligand therapy (RLT) does not prevent patients from either early or eventual disease progression under this treatment. In this study, we investigated co-medication with enzalutamide as a potential re-sensitizer for PSMA-RLT in patients with imminent treatment failure on standard ^177^Lu-based PSMA-RLT. Ten mCRPC patients who exhibited an insufficient response to conventional [^177^Lu]Lu-PSMA-617 RLT received oral medication of enzalutamide 160 mg/d as an adjunct to continued PSMA-RLT. Prostate-specific antigen (PSA) and standard toxicity screening lab work-up were performed to assess the treatment efficacy and safety in these individuals. The mean PSA increase under PSMA-RLT before starting the re-sensitizing procedure was 22.4 ± 26.5%. After the introduction of enzalutamide medication, all patients experienced a PSA decrease, –43.4 ± 20.0% and –48.2 ± 39.0%, after one and two cycles of enzalutamide-augmented PSMA-RLT, respectively. A total of 70% of patients (7/10) experienced partial remission, with a median best PSA response of –62%. Moreover, 5/6 enzalutamide-naïve patients and 2/4 patients who had previously failed enzalutamide exhibited a partial remission. There was no relevant enzalutamide-induced toxicity observed in this small cohort. This pilot experience suggests the synergistic potential of adding enzalutamide to PSMA-RLT derived from the intra-individual comparison of ^177^Lu-based PSMA-RLT ± enzalutamide.

## 1. Background

Advanced prostate cancer under androgen deprivation therapy (ADT) will ultimately result in the stage of metastatic castration-resistant prostate carcinoma (mCRPC) in standard disease evolution [1,2].

Prostate-specific membrane antigen (PSMA)-targeted radioligand therapy (RLT) using PSMA ligands labeled with the beta emitter lutetium-177 (e.g., [^177^Lu]Lu-PSMA-617) has shown encouraging efficacy and safety in patients with mCRPC in various retrospective studies [3,4,5], in prospective phase II trials [6,7] and in a recently published phase III trial (VISION Study) [8]. However, some patients do not respond and some with an initial response will progress during a further course of PSMA-RLT. Thus, there is an unmet need for an enhancement or escalation of the treatment, e.g., by the addition of re-sensitizing medication. Recently, our group reported significant tumoral PSMA upregulation in mCRPC patients by the administration of enzalutamide, a well-established newer androgen axis drug [9], suggesting this agent as a potential (re-)sensitizer for PSMA-RLT [10,11]. In this study, the effect of adding enzalutamide to [^177^Lu]Lu-PSMA-617 RLT in patients with imminent treatment failure is evaluated.

## 2. Methods

In total, n = 10 consecutive mCRPC patients undergoing [^177^Lu]Lu-PSMA-617 RLT with administration of enzalutamide after insufficient response under RLT alone were included in this retrospective study. All patients had exhibited an insufficient response to conventional [^177^Lu]Lu-PSMA-617 RLT with imminent treatment failure before co-medication with enzalutamide was initiated. Insufficient response was defined as any increase or decrease <25% of the prostate-specific antigen (PSA) after the initial cycle(s) of [^177^Lu]Lu-PSMA-617 RLT. All patients had high-volume metastatic disease, and had received various pre-treatments, as presented in Table 1. The median number of PSMA-RLT cycles before adding enzalutamide was 2 (range 1–3), with a median cumulative activity of 10.8 GBq [^177^Lu]Lu-PSMA-617 (range 5.5–17.5 GBq). PSMA-RLT was performed on a compassionate-use basis under the German Pharmaceutical Act §13 (2b). All patients gave their written consent after being thoroughly informed about the risks and side effects, and consented to the publication of their data in accordance with the Declaration of Helsinki. Retrospective analysis was approved by the local Institutional Review Board (ethics committee permission number 140/17).

The co-medication of enzalutamide was prescribed in the well-established standard dosage of 160 mg/d (from a monotherapeutic context [9]), started at beginning of the in-patient stay for PSMA-RLT and maintained at time intervals between consecutive cycles. The radioligand [^177^Lu]Lu-PSMA-617 was intravenously administered during an in-patient stay in accordance with German radiation protection regulations. Regular laboratory assessments, including PSA serum measurements, were analyzed. For the investigation of its potential efficacy, descriptive analysis focused on the PSA course under RLT before and after the initiation of enzalutamide co-medication. Toxicity and adverse events were recorded and graded according to the Common Terminology Criteria for Adverse Events version 5.0 (CTCAE) [12] based on following blood parameters: hemoglobin, leukocytes, platelets, and estimated glomerular filtration rate (eGFR).

## 3. Results

**Figure 1:**
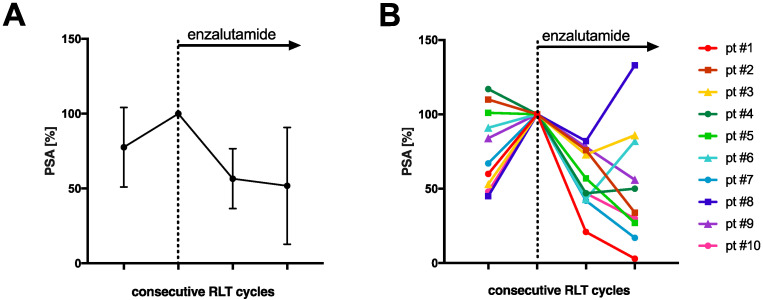
Relative serum PSA course under radioligand therapy prior to (last cycle of conventional PSMA-RLT) and after the start of enzalutamide medication (**A**) for all patients (mean and standard deviation) and (**B**) individually for each patient. PSA was normalized to 100% at start of enzalutamide.

**Figure 2:**
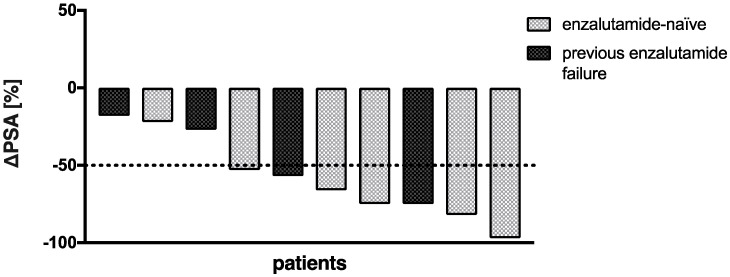
Waterfall plot of best PSA response to PSMA-RLT after start of enzalutamide medication.

**Figure 3:**
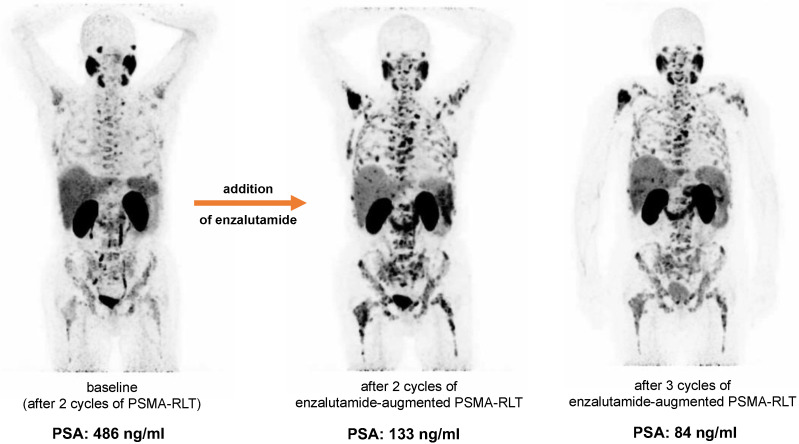
Example of a patient with partial remission after addition of enzalutamide to PSMA-RLT. Increased tumoral uptake after addition of enzalutamide is seen on the maximum intensity projections (MIP) of [^68^Ga]Ga-PSMA-11 PET/CT, while PSA decreases.

The mean PSA increase before initiating co-medication with enzalutamide was 22.4 ± 26.5%. All patients received at least two cycles of enzalutamide-augmented PSMA-RLT (median 5 cycles, range 2–10 cycles) with a median administered per-cycle activity of 6.5 Gbq [^177^Lu]Lu-PSMA-617 (range 2.7–8.5 GBq) continuously after conventional [^177^Lu]Lu-PSMA-617 RLT (5 ± 1 weeks). The mean PSA decrease after one (5 ± 1 weeks) and two cycles (10 ± 2 weeks) of enzalutamide-augmented PSMA-RLT was −43.4 ± 20.0% and −48.2 ± 39.0%, respectively (Figure 1a). The individual PSA courses of each patient are presented in Figure 1b. After initiating co-medication with enzalutamide, all patients experienced a PSA decrease. After two cycles, 6/10 patients showed a further PSA decrease, and 4/10 patients another PSA increase or stable course. In total, 7/10 patients (70%) experienced partial remission defined as serum PSA decrease >50% from the time point before the initiation of enzalutamide (i.e., before the first enzalutamide-enhanced RLT cycle). The median best PSA response was −62% (range −18% to −97%) (Figure 2). Both enzalutamide-naïve patients (5/6 patients) and patients who had previously failed enzalutamide therapy (2/4 patients) exhibited partial remission (Figure 2). The best PSA response was not significantly different in enzalutamide-naïve patients and others (*p* = 0.286, Mann–Whitney U test). An exemplary patient with partial remission is presented in Figure 3.

**Figure 4:**
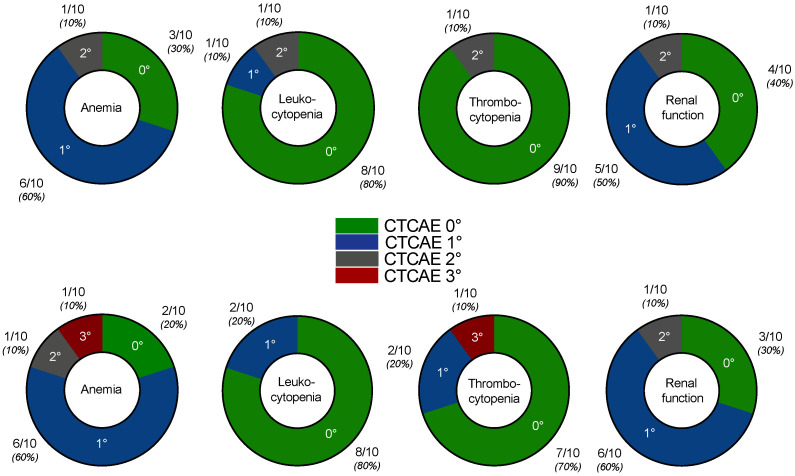
CTCAE grades for anemia, leukocytopenia, thrombocytopenia, and renal function at baseline, i.e., before addition of enzalutamide (**upper row**) and after two cycles of enzalutamide-augmented PSMA-RLT (**lower row**).

No serious acute adverse events were recorded after the addition of enzalutamide. No grade 3/4 toxicities were recorded after two cycles of PSMA-RLT with enzalutamide, with the exception of one patient who had CTCAE 3° anemia and thrombocytopenia. All CTCAE grades for anemia, leukopenia, thrombocytopenia, and renal function impairment before and after two cycles of enzalutamide-augmented PSMA-RLT are shown in Figure 4. The patient who experienced CTCAE 3° anemia and thrombocytopenia had diffuse bone metastases (presented in Figure 3) and was heavily pretreated with multiple regimens of docetaxel and cabazitaxel (re-challenges), ^223^Ra therapy, and abiraterone and presented with CTCAE 2° at baseline. At the end of treatment, no other patient experienced grade 3/4 toxicities.

The median overall survival after the addition of enzalutamide was 12.6 months (95% confidence interval 6.7–18.5 months).

## 4. Discussion

This study suggests the synergistic potential of adding enzalutamide to [^177^Lu]Lu-PSMA-617 RLT, derived from the intraindividual comparison of the pre- and post-enzalutamide initiation disease course under PSMA-RLT in n = 10 treated individuals with mCRPC. Since a significant proportion of otherwise treatment-exhausted patients will experience an insufficient response to [^177^Lu]Lu-PSMA-617 RLT, either early or late during the course of RLT, the improvement of therapeutic efficacy certainly represents a major challenge.

We recently proposed enzalutamide as a potential RLT-enhancing medication (i.e., adjuvant procedure) for boosting PSMA-mediated therapy, describing the significant in vivo upregulation of PSMA expression induced by enzalutamide (re-)exposure, validated by quantitative whole-body [^68^Ga]Ga-PSMA-11 PET/CT measurements [10,11]. There is also increasing evidence from in vitro studies for a significant PSMA-upregulation effect of ADT and androgen axis drugs [13,14,15,16,17,18]. In addition, Emmett et al. and Aggarwal et al. found corresponding systematic effects of enzalutamide on in vivo PSMA PET imaging [19,20]. Our data presented here indicate the first clinical feasibility of adding enzalutamide to patients with insufficient RLT-induced PSA response to achieve a response (with documented partial remission in 7/10 patients (70%) in our cohort). Besides enzalutamide-induced PSMA upregulation, which was also observed in this patient cohort (Figure 3), we think that the general antitumor effect of the androgen receptor cascade blockade is a significant—and probably the major—contributor to the synergistic effect in patients undergoing PSMA-RLT. It should be noted that PSA response was observed in enzalutamide-naïve patients and individuals previously having failed enzalutamide therapy. To rule out the potentially monotherapeutic effect of enzalutamide, a comparison with a control group receiving only enzalutamide (with no accompanying PSMA-RLT) would be necessary.

In contrast to our data, Lückerath et al. did not observe additional tumor growth retardation through the combined enzalutamide/PSMA-RLT treatment in mice bearing CRPC compared to mono-PSMA-RLT, despite significantly greater DNA damage and the persistent increase of PSMA expression [12]. Additional preclinical and clinical studies are certainly needed for further clarification.

After the addition of enzalutamide, we did not observe any effects beyond RLT-related side effects. Thus, based on our small patient cohort, a combination of enzalutamide and PSMA-RLT might be provisionally considered safe. However, this also needs to be investigated in further studies with larger patient cohorts.

The purpose of this communicated experience is to sensitize clinicians to the potential synergistic effect that the addition of enzalutamide may provide to patients undergoing PSMA-RLT. Of course, the reported results should be seen in the light of self-evident limitations. The major limitations are the small patient number (n = 10) and retrospective type of observation. A further major limitation is that a control group of patients with enzalutamide monotherapy is missing. In addition, the applied activity was individually chosen, which may have impacted the treatment outcome. Not all patients received PSMA PET/CT before and after the initiation of enzalutamide, hindering systematic image analyses. Further studies, ideally in a prospective setting with larger patient cohorts, are needed to confirm and expand our findings. Moreover, future studies should also focus on the overall survival of enzalutamide-augmented vs. standard PSMA-RLT.

## 5. Conclusions

The presented pilot experience suggests the synergistic potential of adding enzalutamide to PSMA-RLT, derived from the intra-individual comparison of PSA-response under [^177^Lu]Lu-PSMA-617 ± enzalutamide medication. Controlled studies seem warranted to further investigate this effect.

## Figures and Tables

**Table 1:** Patient characteristics.

Patient Characteristics	Value	
**Age**		
Mean (range) in yrs.	67	(51–88)
≥75 yrs.—n (%)	3	(30)
**PSA**		
Mean (range) in ng/mL	386	(12–1156)
**Sites of metastases—n (%)**		
Bone	8	(80)
Lymph node	9	(90)
Liver	1	(10)
Lung	1	(10)
Other	1	(10)
**Prior treatments—n (%)**		
ADT	10	(100)
Abiraterone or Enzalutamide	9	(90)
Abiraterone	8	(80)
Enzalutamide	4	(40)
Docetaxel or Cabazitaxel	10	(100)
Docetaxel	10	(100)
Cabazitaxel	6	(60)
^223^Ra	1	(10)
**PSMA-RLT before addition of enzalutamide**		
Cycles—median (range)	2	(1–3)
Cum. Activity—median (range) in GBq	10.8	(5.5–17.5)

## Data Availability

The datasets used and analyzed during the current study are available from the corresponding author on reasonable request.
